# Regression of macular edema with topical brinzolamide and nepafenac alone and identification of a novel gyrate atrophy mutation

**DOI:** 10.5935/0004-2749.20200028

**Published:** 2020

**Authors:** Cemal Çavdarlı, Esra Şahlı, Büşranur Çavdarlı, Mehmet Numan Alp

**Affiliations:** 1 Department of Ophthalmology, Ankara Numune Training and Research Hospital, University of Health Sciences, Ankara, Turkey; 2 Department of Medical Genetics, Ankara Numune Training and Research Hospital, University of Health Sciences, Ankara, Turkey

**Keywords:** Brinzolamide, Macular edema, Genes, Mutation, Nepafenac, Ornithine, Transaminases, Brinzolamida, Edema macular, Genes, Mutação, Ne pafenaco, Ornitina, Transaminases

## Abstract

Gyrate atrophy is a rare metabolic autosomal recessive disorder caused by ornithine aminotransferase enzyme deficiency that leads to characteristic progressive, degenerative chorioretinal findings. Patients complain mostly of low vision, night blindness, and peripheral vision loss. Posterior subcapsular cataract, myopia, choroid neovascularization, and intraretinal cysts may be accompanying factors related to vision loss. We encountered a patient with vision loss secondary to posterior subcapsular cataract and intraretinal cysts. After treatment with topical brinzolamide and nepafenac (and without any diet mo dification and/or supplementation), we observed 143- and 117-µm macular thickness resolutions with 2 and 1 Snellen lines of visual gain in his right and left eyes, respectively. Also, we detected a novel homozygous mutation in the ornithine aminotransferase gene: c.1253T>C (p.Leu418Pro). Carbonic anhydrase inhibitors and/or non-steroid anti-inflammatory drugs can control macular edema in patients with gyrate atrophy-associated intraretinal cysts. The genetic variants may also be a determinant in the responsiveness to the therapy type.

## Abstract (pt)

A atrofia girata é um distúrbio autossômico recessivo metabólico raro causado pela deficiência da enzima ornitina ami notransferase, que leva a achados degenerativos coriorretinianos progressivos característicos. Os pacientes queixam-se principalmente de baixa visão, cegueira noturna e perda de vis ão periférica. A catarata subcapsular posterior, a miopia, a neovascularização da coróide e os cistos intrarretinianos podem ser fatores associados à perda da visão. Encontramos um paciente com perda de visão secundária à catarata subcapsular posterior e cistos intrarretinianos. Após o tratamento com brinzolamida tópica e nepafenaco (e sem modificação e/ou suplementação da dieta), observamos resoluções de espessura macular de 143 e 117 µm e com 2 e 1 linhas de Snellen de ganho visual nos olhos direito e esquerdo, respectivamente. Além disso, detectamos uma nova mutação homozigótica no gene da ornitina aminotransferas e: c.1253T>C (p.Leu418Pro). Inibidores da anidrase carbônica e/ ou drogas anti-inflamatórias não esteróides podem controlar o edema macular em pacientes com cistos intrarretinianos associados à atrofia girata. As variantes genéticas também podem ser determinantes na responsividade ao tipo de terapia.

## INTRODUCTION

Gyrate atrophy (GA) is a rare metabolic disorder of autosomal recessive inheritance due to ornithine aminotransferase (OAT) enzyme deficiency that causes characteristic chorioretinal findings. The OAT enzyme encoded by the homonymous gene located on 10q26.13 with ten functional exons is a pyridoxal phosphate (vitamin B6)-dependent mitochondrial enzyme and catalyzes the conversion of ornithine into glutamic acid and proline^(1)^. Enzyme deficiencies caused by mutations in the OAT gene result in high plasma ornithine concentrations and the progression of chorioretinal lesions in GA; an arginine-restricted diet is thought to reduce the plasma ornithine levels^(2)^. GA is a progressive, degenerative chorioretinal disorder accompanied by funduscopic findings that include sharp demarcated circular chorioretinal atrophic areas observed mainly in the periphery of the retina. With age, these lesions generally increase in number and size and involve the posterior pole of the retina^(3,4)^. The main patient complaints are low vision, peripheral vision loss, and night blindness during the second decade of life. Posterior subcapsular cataract, myopia, choroid neovascularizations, and intraretinal cysts (ICs)^(4,5)^may be causal factors of vision loss. In this report, we present the case of a 26-year-old man with GA and a novel OAT mutation, and report on the regression of his ICs after topical brinzolamide 1% with nepafenac 0.1% eye drops.

## CASE REPORT

**Figure 1:**
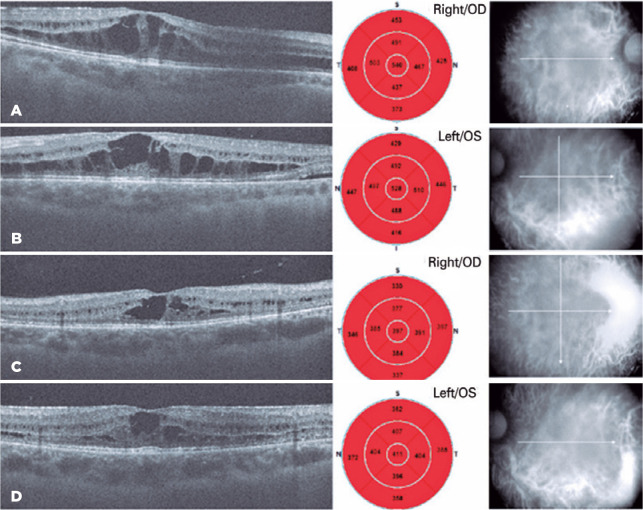
Bilateral spectral domain optical coherence tomography (sd-OCT) central foveolar cystoid macular edema at the initial visit (A, B). Two months later at the second visit, sd-OCT of central foveolar scans show macular edema regression (C, D).

**Figure 2:**
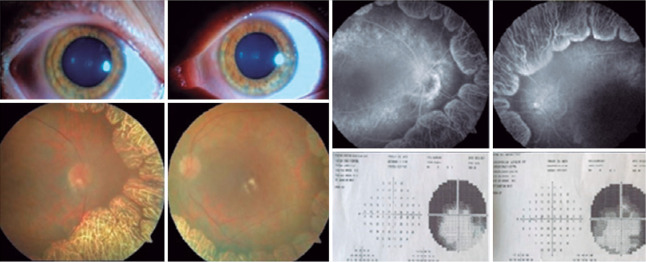
View of anterior segment photography, fundus photography, fluorescein angiography, and visual field (Humphrey visual field, Zeiss, Oberkochen, Germany).

A 26-year-old man was referred to our clinic with a complaint of progressive vision loss over one year. We performed a complete ophthalmologic examination including a visual field analysis. His manifest auto-refractions were -0.25 -4.75 ∞160° and -1.00 -4.50∞20° within his right and left eyes, respectively. The Snellen best-corrected visual acuity (BCVA) was 20/100 in both eyes. His anterior segment and dilated funduscopic examination revealed bilateral posterior subcapsular cataracts and peripheral-midperipheral sharp demarcated circular chorioretinal atrophic areas (Figure 1). His intraocular pressures were in the normal range (12 and 13 mmHg, respectively). We also performed retinal spectral domain optical coherence tomography (SD-OCT, Cirrus HD-OCT 5000, Carl Zeiss Meditec, Jena, Germany), and we detected bilateral IC-like cystoid macular edema (Figure 1). The central macular thicknesses were 540 µm in his right eye and 528 µm in his left eye. In addition, we performed fundus photography and fluorescein angiography and observed peripheral and midperipheral window defects due to chorioretinal atrophic lesions (Figure 2).

We suspected the presence of GA based on the eye examinations and decided to revisit the anamnesis. We learned that the patient had been prescribed an arginine-restricted diet since 2008 to lower his ornithine levels, but he had not paid much attention to do it. We examined his blood and urine reports from that time. The plasma and urine ornithine levels were 379 µmol/L and 9363.7 µmol/g creatinine, respectively. After obtaining this evidence for GA, we decided to start topical brinzolamide 1% with nepafenac 0.1% as a combination therapy for his macular edema^(6)^ (Figures 1A, 1B). We also requested consultations for him at the department of endocrinology and metabolism and with a nutritionist to help him achieve metabolic control and diet adaptation. Also, we referred him to the genetic counseling clinic at the Department of Medical Genetics for Molecular Analyses and Genetic Counselling.

**Figure 3:**
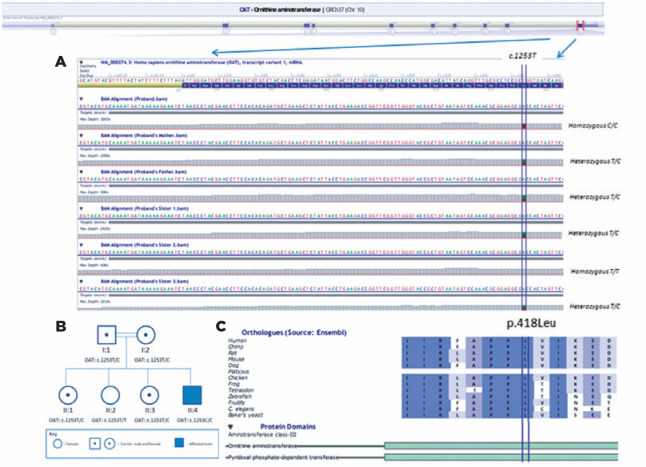
(A) Next-generation sequencing (NGS) alignment of the patient, parents, and sisters in the Binary Alignment Map (BAM) format. Transcript region of c.1253, the proband’s sequence alignment, the proband’s father’s sequence alignment, the proband’s mother’s sequence alignment, and the proband’s sister’s alignments are shown, respectively. (B) Pedigree and genotypes of the proband’s family. (C) Orthologues in 10 species and protein domains.

After isolation of genomic DNA, the OAT gene was sequenced using a next-generation sequencing platform (Mysec-Illumina, San Diego, CA, USA). Libraries were pre pared with the NextEra XT kit (Illumina, San Diego, CA, USA). Sequence alignments were performed according to the hg19 genome within the MiSeq Reporter software (Illumina) and visualized with the ALAMUT^®^ VISUAL software (Interactive Biosoftware, city, France). A novel homozygous mutation was detected in the OAT gene: c.1253T>C (p.Leu418Pro). All the family members were investigated for the same mutation (Figures 3A, 3B present the results).

We reexamined the patient two months after his first visit. According to the anamnesis, he had used the given topical medications, regularly. However, he was not adapted completely to the given diet. His Snellen BCVAs were 20/50 and 20/63 in the right and left eyes, respectively, and the ICs on SD-OCT had regressed (Figures 1C, 1D). His final central macular thicknesses were 397 µm in his right eye and 411 µm in his left eye.

## DISCUSSION

GA is a rare inherited autosomal recessive progressive chorioretinal dystrophy associated with high plasma ornithine levels secondary to vitamin B6-dependent OAT enzyme deficiency; the associated intraretinal fluid or cysts are the leading causes of central vision impairment in these patients^(4)^. The disruption of the outer blood-retinal barrier due to retinal pigment epithelial dysfunction in retinal dystrophies is thought to be responsible for the diffusion of fluid into the intraretinal spaces^(7)^. Tangential vitreous forces, disruption of the retinal cell-to-cell adhesion, and retinal pigment epithelium pumping failure have also been considered as potential causes of the visual impairment^(4)^. An arginine-restricted diet, vitamin B6 supplementation, carbonic anhydrase inhibitors (CAI), and topical non-steroid anti-inflammatory drugs (NSAIDs) have all been used to treat ICs in GA^(6)^, and all these approaches have been effective. Although we tried to help our patient adapt to his diet, we failed. We prescribed a topical CAI with a NSAID bilaterally to resolve the macular edema as recommended^(6)^. Two months after his initial visit and on his topical treatment, the patient had gained 2 and 1 Snellen lines of visual acuity, and 143- and 117-µm IC resolutions in his right and left eyes, respectively. We believe our experience with this patient is important because it highlights the successful therapy with topical CAIs with NSAIDs, despite the noncompliance with the recommended diet. Additionally, we detected a homozygous c.1253T>C (p.Leu418Pro) variant in our patient, and the heterozygous state in consanguineous parents and in two unaffected sisters. A clinically normal sister had the wild-type genotype (Figures 3A, 3B). In the OAT gene, 108 variants have been classified as “pathogenic” and “likely pathogenic” in an up-to-date Clinvar database by the literature and expert diagnostic laboratories. We found that 39 of 108 variants were missense as those detected in our patient. The “c.1253T>C” variant had not been identified, and the allele was not found in the GnomAD exome and GnomAD genome projects^(8)^. *In silico* prediction tools suggested the variant as deleterious and decreed that it has a damaging effect on protein function. A leucine aminoacid in position 418 of the protein is located at a functional domain and is highly conserved among the species (Figure 3C). Thus, we interpreted the mutation as likely pathogenic according to the criterias of the ACMG 2015 Guidelines^(9)^.

In conclusion, patients usually benefit from arginine-restricted and low-protein diets in terms of IC reductions. However, in some cases, ICs can be resistant to diet^(10)^, or patients cannot adapt to their diets as re quired. Also, results of the vitamin B6 supplementation are variable in the literature. CAIs and/or NSAIDs can be useful to control ICs in such cases. The genetic mutation variants may also be a determinant in the responsiveness to each type of therapy, but we need more genotype-phenotype studies to confirm this.

## References

[1] Ginguay A, Cynober L, Curis E, Nicolis I. (2017). Ornithine aminotransferase, an important glutamate-metabolizing
enzyme at the crossroads of multiple metabolic pathways. Biology (Basel).

[2] Kaiser-Kupfer MI, Caruso RC, Valle D. (2002). Gyrate atrophy of the choroid and retina: further experience with
long-term reduction of ornithine levels in children. Arch Ophthalmol.

[3] Sergouniotis PI, Davidson AE, Lenassi E, Devery SR, Moore AT, Webster AR. (2012). Retinal structure, function, and molecular pathologic features in
gyrate atrophy. Ophthalmology.

[4] Salvatore S, Fishman GA, Genead MA. (2013). Treatment of cystic macular lesions in hereditary retinal
dystrophies. Surv Ophthalmol.

[5] Takki KK, Milton RC. (1981). The natural history of gyrate atrophy of the choroid and
retina. Ophthalmology.

[6] Piozzi E, Alessi S, Santambrogio S, Cillino G, Mazza M, Iggui A (2017). Carbonic anhydrase inhibitor with topical NSAID therapy to manage
cystoid macular edema in a case of gyrate atrophy. Eur J Ophthalmol.

[7] Oliveira TL, Andrade RE, Muccioli C, Sallum J, Belfort R Jr. (2005). Cystoid macular edema in gyrate atrophy of the choroid and
retina: a fluorescein angiography and optical coherence tomography
evaluation. Am J Ophthalmol.

[8] Lek M, Karczewski KJ, Minikel EV, Samocha KE, Banks E, Fennell T (2016). Exome Aggregation Consortium. Analysis of protein-coding genetic
variation in 60706 humans. Nature.

[9] Richards S, Aziz N, Bale S, Bick D, Das S, Gastier-Foster J (2015). ACMG Laboratory Quality Assurance Committee Standards and
guidelines for the interpretation of sequence variants: a joint consensus
recommendation of the American College of Medical Genetics and Genomics and
the Association for Molecular Pathology. Genet Med.

[10] Doguizi S, Sekeroglu MA, Anayol MA, Yilmazbas P. (2015). Arginine-restricted therapy resistant bilateral macular edema
associated with gyrate atrophy. Case Rep Ophthalmol Med.

